# Possible Novel Therapy for Malignant Gliomas with Secretable Trimeric TRAIL

**DOI:** 10.1371/journal.pone.0004545

**Published:** 2009-02-20

**Authors:** Moonsup Jeong, Yong-Sam Kwon, Soon-Hye Park, Chae-Young Kim, Sin-Soo Jeun, Kang-Won Song, Yong Ko, Paul D. Robbins, Timothy R. Billiar, Byong-Moon Kim, Dai-Wu Seol

**Affiliations:** 1 Biopharmaceutical Research Laboratories of Dong-A Pharmaceutical Co., Ltd., Kyunggi-Do, Korea; 2 Department of Neurosurgery, The Catholic University of Korea, Seoul, Korea; 3 Department of Neurosurgery, Hanyang University Medical Center, Seoul, Korea; 4 Department of Molecular Genetics and Biochemistry, University of Pittsburgh School of Medicine, Pittsburgh, Pennsylvania, United States of America; 5 Department of Surgery, University of Pittsburgh School of Medicine, Pittsburgh, Pennsylvania, United States of America; 6 Department of Life Science, Kyungwon University, Kyunggi-Do, Korea; 7 Gachon BioNano Research Institute, Kyungwon University, Kyunggi-Do, Korea; National Cancer Institute at Frederick, United States of America

## Abstract

Malignant gliomas are the most common primary brain tumors. Despite intensive clinical investigation and many novel therapeutic approaches, average survival for the patients with malignant gliomas is only about 1 year. Tumor necrosis factor-related apoptosis-inducing ligand (TRAIL) has shown potent and cancer-selective killing activity and drawn considerable attention as a promising therapy for cancers, but concerns over delivery and toxicity have limited progress. We have developed a secretable trimeric TRAIL (stTRAIL) and here evaluated the therapeutic potential of this stTRAIL-based gene therapy in brain tumors. An adenovirus (Ad-stTRAIL) delivering stTRAIL was injected into intra-cranial human glioma tumors established in nude mice and tumor growth monitored using the magnetic resonance imaging (MRI). Ad-stTRAIL gene therapy showed potent tumor suppressor activity with no toxic side effects at therapeutically effective doses. When compared with 1, 3-bis(2-chloroethyl)-1-nitrosourea (BCNU), a conventional therapy for malignant gliomas, Ad-stTRAIL suppressed tumor growth more potently. The combination of Ad-stTRAIL and BCNU significantly increased survival compared to the control mice or mice receiving Ad-stTRAIL alone. Our data indicate that Ad-stTRAIL, either alone or combined with BCNU, has promise as a novel therapy for malignant gliomas.

## Introduction

Tumor necrosis factor (TNF)-related apoptosis-inducing ligand (TRAIL) is a member of the TNF family [1], [2]. TRAIL is primarily expressed as a type II transmembrane protein in which the carboxyl terminus of the receptor-binding domain protrudes extracellularly. Structural studies have demonstrated that biologically active soluble TRAIL forms a homotrimer [3], [4]. This homotrimeric structure of TRAIL is stabilized by a cysteine residue at position 230 that coordinates with a divalent zinc ion [4]–[6]. As the depletion of the zinc ion or a mutation of the cysteine residue to alanine or glycine abrogates functional activity of TRAIL [5], [6], homotrimerization of TRAIL is pivotal for its biological activity.

TRAIL acts through binding to its cognate receptors DR4 and/or DR5 [7]–[9]. Once activated, DR4 and/or DR5 transmit apoptotic signals intracellularly. Similar to other TNF family members such as FasL and TNF-a, TRAIL activates caspase-8 as an initial apoptotic signaling event [10], [11]. Despite similarity in apoptotic signaling cascades activated but the mechanism largely unknown yet, TRAIL has a unique selectivity for triggering apoptosis in tumor cells but not most normal cells [12], [13]. In addition, complying with the tissue distribution of the TRAIL receptors, TRAIL has a wide range of targets. These features of TRAIL have drawn considerable attention as a promising cancer therapy [14].

Malignant gliomas are the most common brain tumors in adults and account for more than half of all brain tumors [15], [16]. However, treatment of malignant gliomas with conventional approaches is largely unsuccessful because a wide resection commonly applied for other malignancies is limited and curative doses of therapeutics generally cannot be delivered to the tumor site without excessive toxicity to normal tissues. As a consequence, the mortality rate for the patients harboring malignant gliomas remains high for the past decades. Here, we present a novel TRAIL-based gene therapy for malignant gliomas. Our gene therapy approach showed potent tumor suppressor activity for intracranial brain tumor with no toxic side effects. Our data indicate that this approach has great promise as a therapy toward malignant gliomas.

## Results and Discussion

**Figure 1:**
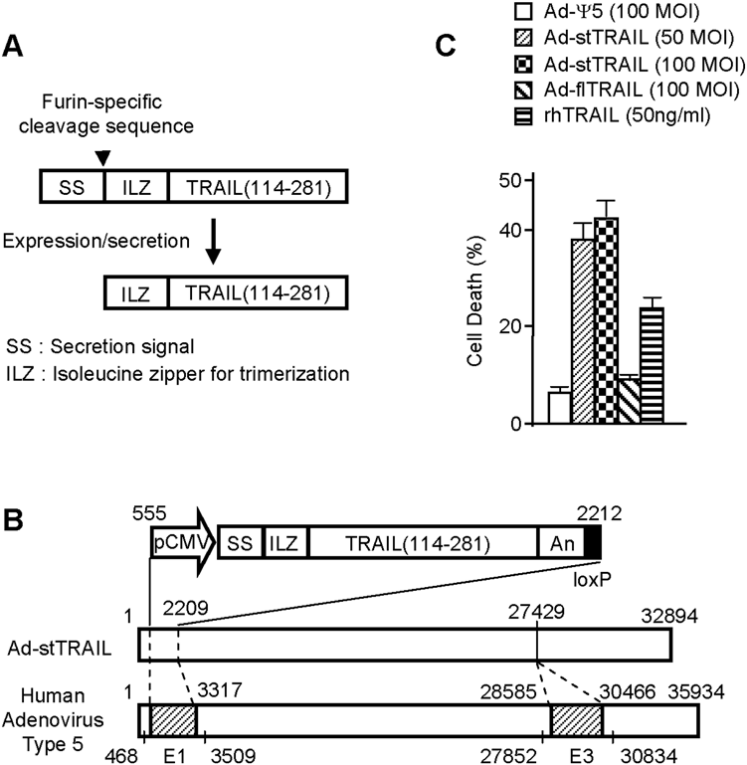
Construction of Ad-stTRAIL and Tests of stTRAIL Secretion. (A) Schematic drawing of the stTRAIL expression cassette. (B) Schematic drawing for constructing Ad-stTRAIL. An E1/E3-doubly deleted replication-incompetent adenoviral vector Ad-stTRAIL was constructed through Cre-lox recombination. (C) Trans-well analysis. U373-MG cells were infected with adenovirus for 6 hours, washed two times with HBS and transferred to the upper wells. U87-MG cells maintained at the bottom wells were co-cultured for 3 days with the upper wells and subjected to cell viability assay. rhTRAIL was used as a positive control. The results represent the mean and SE for five separate experiments.

TRAIL is a membrane-bound ligand [1], [2] which acts through its cognate death receptors DR4 and/or DR5 on the surface of target cells [7]–[9], [14]. It is thus expected that secreted soluble TRAIL should kill a larger number of cells through wider distribution. To develop a biologically active form of soluble TRAIL produced in mammalian cells, we manipulated the apoptosis-inducing moiety of TRAIL (amino acids 114–281) to be secreted through fusion with a secretion signal sequence. Interestingly, however, we observed that unlike recombinant soluble TRAIL (114–281) produced from bacterial cells, the secreted TRAIL (114–281) produced in mammalian cells maintains its apoptotic functionality only when it is fused with a trimerization-enforcing domain [17]. This result suggested that the soluble TRAIL (114–281) ectopically expressed in mammalian cells does not form the trimer quaternary structure required for apoptotic activity [3], [4], [6]. Based on these observations, we have created an expression cassette that encodes a secretable form of trimeric TRAIL (stTRAIL) composed of the three functional elements including a secretion signal, a trimerization domain and an apoptosis-inducing moiety of TRAIL gene sequence (Figure 1A). The stTRAIL is expressed as a pro-protein (SS-ILZ-TRAIL) and converted to an active protein (ILZ-TRAIL) following a specific cleavage by Furin [18], a protease residing in Golgi complex, in the process of secretion.

In an attempt to establish a gene therapy approach to deliver stTRAIL to tissues efficiently, we inserted our stTRAIL expression cassette to Ψ5 [19], an E1/E3- doubly deleted human adenovirus type 5 (Figure 1B). We compared apoptotic activity of Ad-stTRAIL with that of an adenoviral vector (Ad-flTRAIL) carrying membrane-bound full-length TRAIL (flTRAIL) in a trans-well system that allows only soluble and diffusible agents to reach target cells. Ad-flTRAIL showed no observable cell death, whereas Ad-stTRAIL effectively induced death in the U87-MG target cells (Figure 1C). Ad-stTRAIL also induced higher apoptotic activity than Ad-flTRAIL (data not shown). Our data indicate that stTRAIL is diffusible and induces death in cells remote from the site of production whereas membrane-bound full-length TRAIL does not.

**Figure 2:**
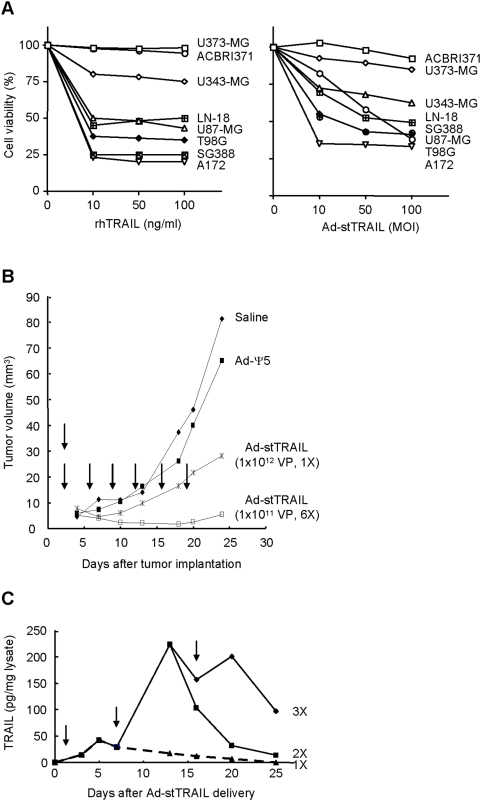
In vitro and in vivo Tumoricidal Activity Assessment of Ad-stTRAIL. (A) Glioma cells, plated in 24-wells, were treated with rhTRAIL (0∼100 ng/ml) or Ad-stTRAIL (1∼100 MOI) and incubated for 48 h, followed by cell viability analysis as described in *Materials and Methods*. The results represent the mean for five separate experiments. The SE bars were omitted, but were <5% at all the data points. (B) Better tumor suppressor activity by divided low-dosed multiple delivery of Ad-stTRAIL. 1×10^6^ U87-MG cell were implanted to the left flank of 6-week-old SCID mice (Jackson Laboratory) and treated with saline control or different modalities. To compare a single high-dose injection versus divided low-dosed multiple injections in tumor suppressor activity, Ad-stTRAIL was delivered as indicated by arrows (n = 5 for each experimental group; total n = 20). The results represent the mean for the tumor volume. The SE bars were omitted, but were <5% at all the data points. There were statistically significant difference between any of two groups (*P*<0.05). (C) The expression profile of stTRAIL protein by multiple delivery of Ad-stTRAIL. When the tumor diameter reached 6∼8 mm after implanting U87-MG cells as described in (A), Ad-stTRAIL (1×10^12^ VP) was delivered once, twice or three times at 7-day intervals. Tumor tissues were isolated and subjected to analysis of stTRAIL protein. The results represent the mean from three mice used for each experimental group. The SE bars were omitted, but were <5% at all the data points.

We then evaluated the potential of Ad-stTRAIL as a therapeutic for gliomas *in vitro* and *in vivo*. In tests using various glioma cells (Figure 2A), Ad-stTRAIL induced cell death in all except U373-MG and ACBRI371, both of which also showed significant resistance to recombinant TRAIL protein. Despite their resistance to TRAIL (recombinant TRAIL protein or Ad-stTRAIL), U373-MG and ACBRI371 cells expressed TRAIL receptors DR4 and DR5 (data not shown). Moreover, all the cell lines also well expressed CAR, an adenovirus receptor molecule. Thus, resistance of U373-MG and ACBRI371 to Ad-stTRAIL seems unlikely to be associated with efficiency of adenovirus infection. Currently, resistance of U373-MG cells to TRAIL is under investigation.

In an animal tumor model established by subcutaneous injection of U87-MG cells derived from human glioblastoma multiform, Ad-stTRAIL (single injection of 1×10^12^ or six injections of 1×10^11^ virus particles) also significantly suppressed tumor growth (Figure 2B). The six injections more effectively suppressed tumor growth than the single injection using a higher dose. In fact, multiple delivery of Ad-stTRAIL to tumors resulted in sustained increase in stTRAIL levels in the tumor injection site (Figure 2C).

**Figure 3:**
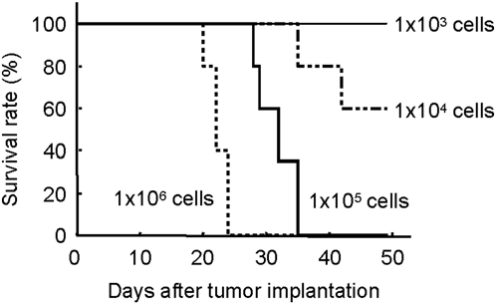
Establishment of an Animal Brain Tumor Model. Various number of U87-MG cells were stereotactically implanted to the right striatum of the athymic mice (n = 6 for each experimental group; total n = 24). A burr hole was drilled in the skull lateral to the bregma and Hamilton syringe (22 G) was introduced to a depth of 3 mm. Survival was examined and plotted.

Based on these promising results, we then sought to determine the therapeutic efficacy of Ad-stTRAIL in a murine glioma model. To establish a human glioma model, nude mice were injected intra-cranially with varying numbers of U87-MG cells. We identified that injection of 1×10^5^ cells/brain produces the most appropriate animal model in terms of tumor growth, size and animal longevity (Figure 3). Thus, throughout our studies to evaluate therapeutic efficacy of Ad-stTRAIL, we used this animal brain tumor model.

**Figure 4:**
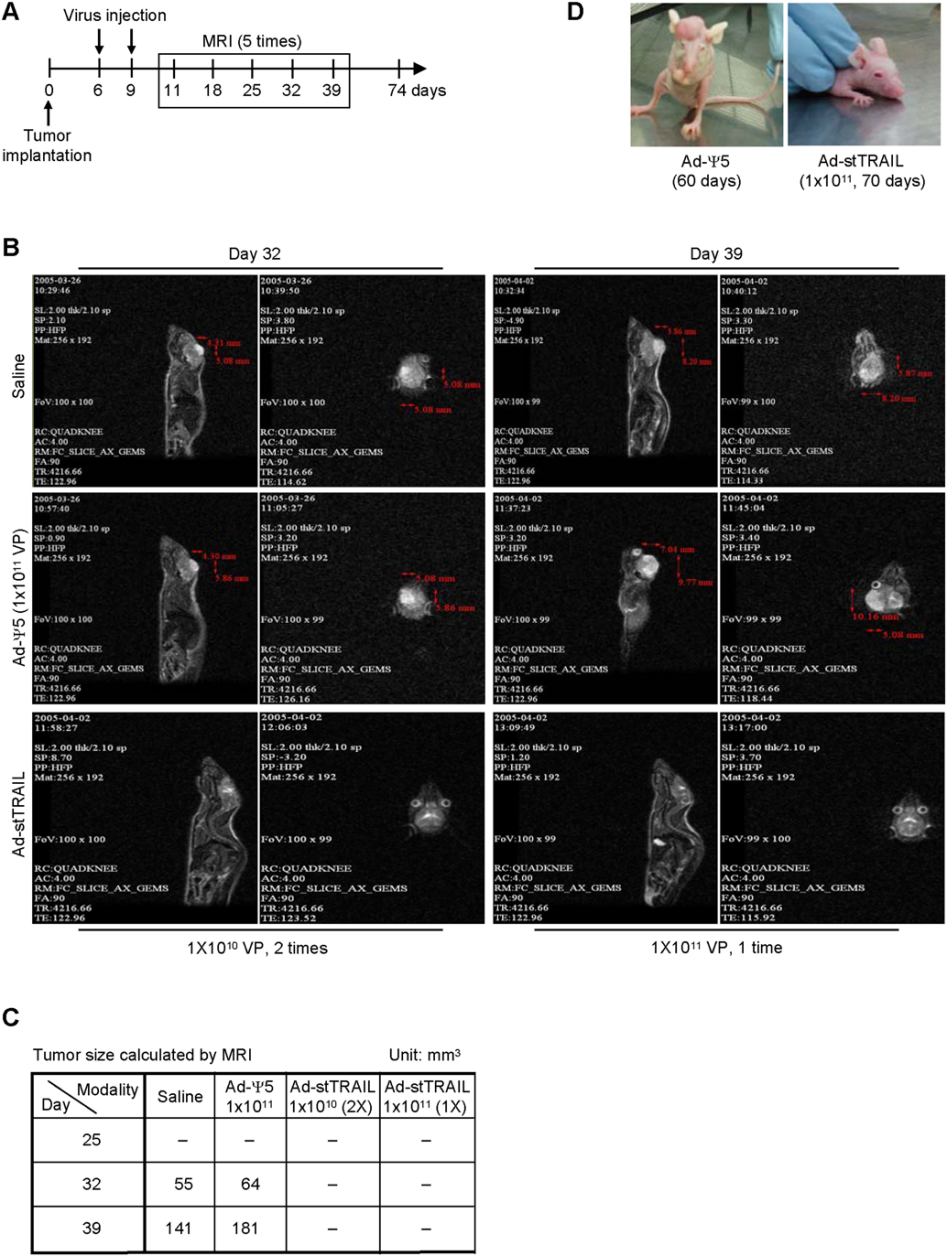
Evaluation of Ad-stTRAIL as a Therapy for Gliomas. (A, B, C) The schematic drawing for experimental design and MRI analysis. U87-MG human malignant glioma cells (1×10^5^ cells/10 µL per mouse brain) were stereotactically implanted into the right striatum of athymic mice (nu/nu). 6 and 10 days after tumor implantation, controls (saline or Ad-Ψ5) or Ad-stTRAIL were delivered intra-tumorally. Tumor growth was monitored by MRI at the indicated times. (D) Representative animals treated with Ad-Ψ5 or Ad-stTRAIL.

First, to assess therapeutic efficacy of Ad-stTRAIL, we delivered Ad-stTRAIL at day 6 and 9 into the tumor site and monitored tumor growth in live animals using MRI (Figure 4A, B, C). MRI analysis showed no tumor growth in either the control or the modality-treated groups during first 25 days. At day 32, however, the control groups (saline and Ad-Ψ5) showed significant tumor growth, while the modality-treated groups exhibited no growth. In fact, even at day 70 after the delivery of Ad-stTRAIL, no tumors were detectable in the modality-treated groups (Figure 4D).

**Figure 5:**
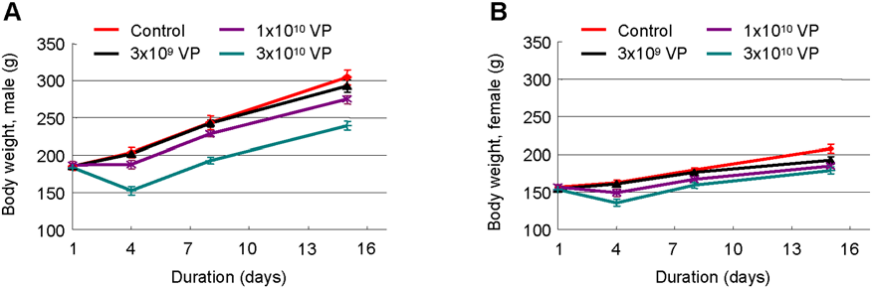
Safety Assessment of Ad-stTRAIL in Animals. (A, B) Body weight changes in rats. The control (virus formulation buffer) or the modality (3×10^9^, 1×10^10^ or 3×10^10^ viral particles) was delivered to the putamen region of rat brain and body weights were measured at the indicated times (n = 6 for each experimental group; total n = 48). The results represent the mean and SE.

Next, we evaluated side effects of Ad-stTRAIL in animals. We followed the guideline of Korea Food and Drug Administration (KFDA) for the development of new drugs. Accordingly, we used normal rats and measured side effects after injecting various doses of Ad-stTRAIL to the putamen region of rat brain. We first determined the lethal dose of Ad-stTRAIL. As shown in Table 1, rats tolerated all doses of Ad-stTRAIL tested. Therefore, the lethal intracranial dose in rats is higher than 3×10^10^ viral particles per a single injection. No change in body weight over time was observed with injections of 3×10^9^ and 1×10^10^ viral particles, while the highest dose gave rise to a transient decline in body weight (Figure 5), which recovered after one week in both male and female rats. Delivery of the same doses of Ad-stTRAIL to the brains of normal mice did not significantly affect body weight (data not shown). More importantly, when the same doses of viral particles were directly delivered to tumors implanted in brains of mice, no apparent side effects, including loss of body weight, decreased food and water consumption, coughing, nasal discharge and hunched posture, were observed (data not shown). Our data indicate that administration of therapeutic doses of Ad-stTRAIL injected into either normal brains or brain tumors is safe with no apparent side effects.

**Table 1:** Mortality and Determination of the Lethal Dose.

Sex	Group/Dose (VP/head)	No. of animals	Day after treatment															Mortality (dead/total)	Approximate lethal dose (VP/head)
			1	2	3	4	5	6	7	8	9	10	11	12	13	14	15		
Male	Control	6	0	0	0	0	0	0	0	0	0	0	0	0	0	0	0	0% (0/6)	Higher than 3×10^10^
	3×10^9^	6	0	0	0	0	0	0	0	0	0	0	0	0	0	0	0	0% (0/6)	
	1×10^10^	6	0	0	0	0	0	0	0	0	0	0	0	0	0	0	0	0% (0/6)	
	3×10^10^	6	0	0	0	0	0	0	0	0	0	0	0	0	0	0	0	0% (0/6)	
Female	Control	6	0	0	0	0	0	0	0	0	0	0	0	0	0	0	0	0% (0/6)	Higher than 3×10^10^
	3×10^9^	6	0	0	0	0	0	0	0	0	0	0	0	0	0	0	0	0% (0/6)	
	1×10^10^	6	0	0	0	0	0	0	0	0	0	0	0	0	0	0	0	0% (0/6)	
	3×10^10^	6	0	0	0	0	0	0	0	0	0	0	0	0	0	0	0	0% (0/6)	

The control (the formulation buffer) or the modality (3×10^9^, 1×10^10^ or 3×10^10^ viral particles) at 30 µl/head was delivered to the putamen region of rat brain as described in *Materials and Methods* and mortality was examined.

**Figure 6:**
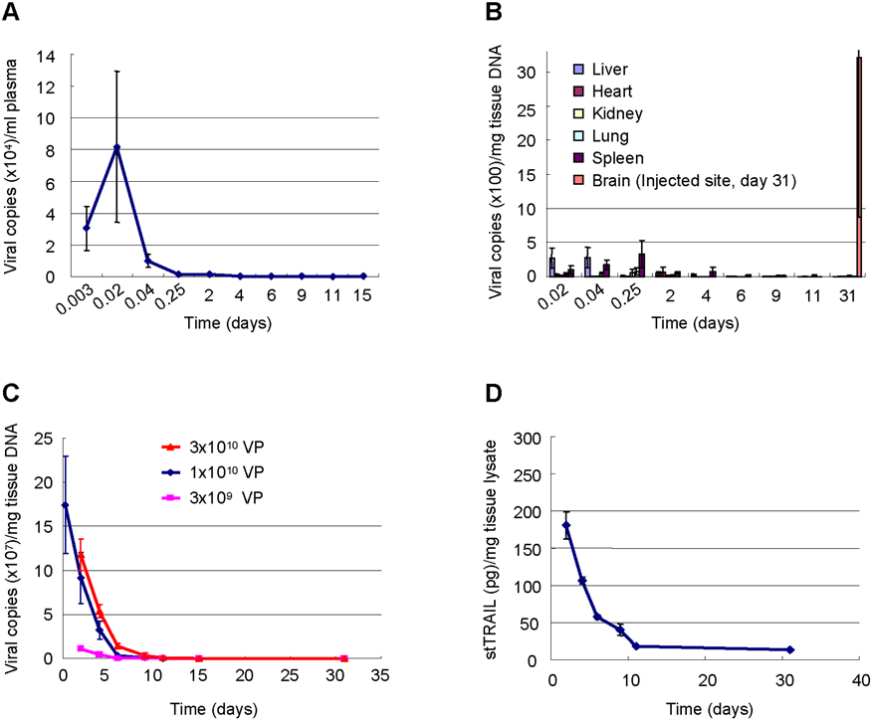
Tissue Distribution of Ad-stTRAIL in Animals. (A, B) Ad-stTRAIL (1×10^10^ viral particles) was delivered to the putamen region of rat brain and subjected to analysis at the indicated times for Ad-stTRAIL DNA copies in blood plasma (A) and in different organs (B). The results represent the mean and SE for six separate experiments. (C) Ad-stTRAIL (3×10^9^, 1×10^10^ or 3×10^10^ viral particles) was delivered to the putamen region of rat brain and analyzed at the indicated times for Ad-stTRAIL DNA copies in the injection site. The results represent the mean and SE for six separate experiments. (D) After delivery of Ad-stTRAIL (1×10^10^ viral particles) to the putamen region of rat brain as described in (B), stTRAIL protein in the injection site was analyzed at the indicated times. The results represent the mean and SE for six separate experiments.

To avoid any unwanted systemic toxicity, we consider only intra-tumoral delivery of Ad-stTRAIL as a potential therapeutic approach. It is important to know if the intra-cranially delivered viral particles cross the blood-brain barrier to enter the blood circulation resulting in possible systemic exposure. To address this question, we analyzed viral DNA in blood plasma and multiple organs at different time points following delivery of Ad-stTRAIL (1×10^10^ viral particles) to rat brain. Detectable DNA peaked in the blood at 30 min and then rapidly disappeared (Figure 6A). Similarly, viral DNA was detectable in organs during only early time points (Figure 6B). Although viral DNA was detected in the blood circulation and organs, it is important to point that the amount of viral DNA measured outside of the brain, even at the peak, was just 0.0008% of the total input. Minimal stTRAIL protein was detected in the blood plasma and liver (data not shown). Thus, the quantity of viral particles and recombinant protein that transiently enters the systemic circulation after brain injection appears to be very limited. In contrast, viral DNA at the injection site persisted longer (Figure 6C) and both DNA and protein at the injection site correlated with the dose of viral particles (Figure 6D). In addition to the blood plasma, we also examined the viral particles in circulating blood cells including red blood cells and lymphocytes. No significant amount of viral DNA was observable in these cells (data not shown). Our data indicate that Ad-stTRAIL cannot freely cross the blood-brain barrier and remains localized mainly at the delivery site, where the expression is sustained without systemic side effects.

**Figure 7:**
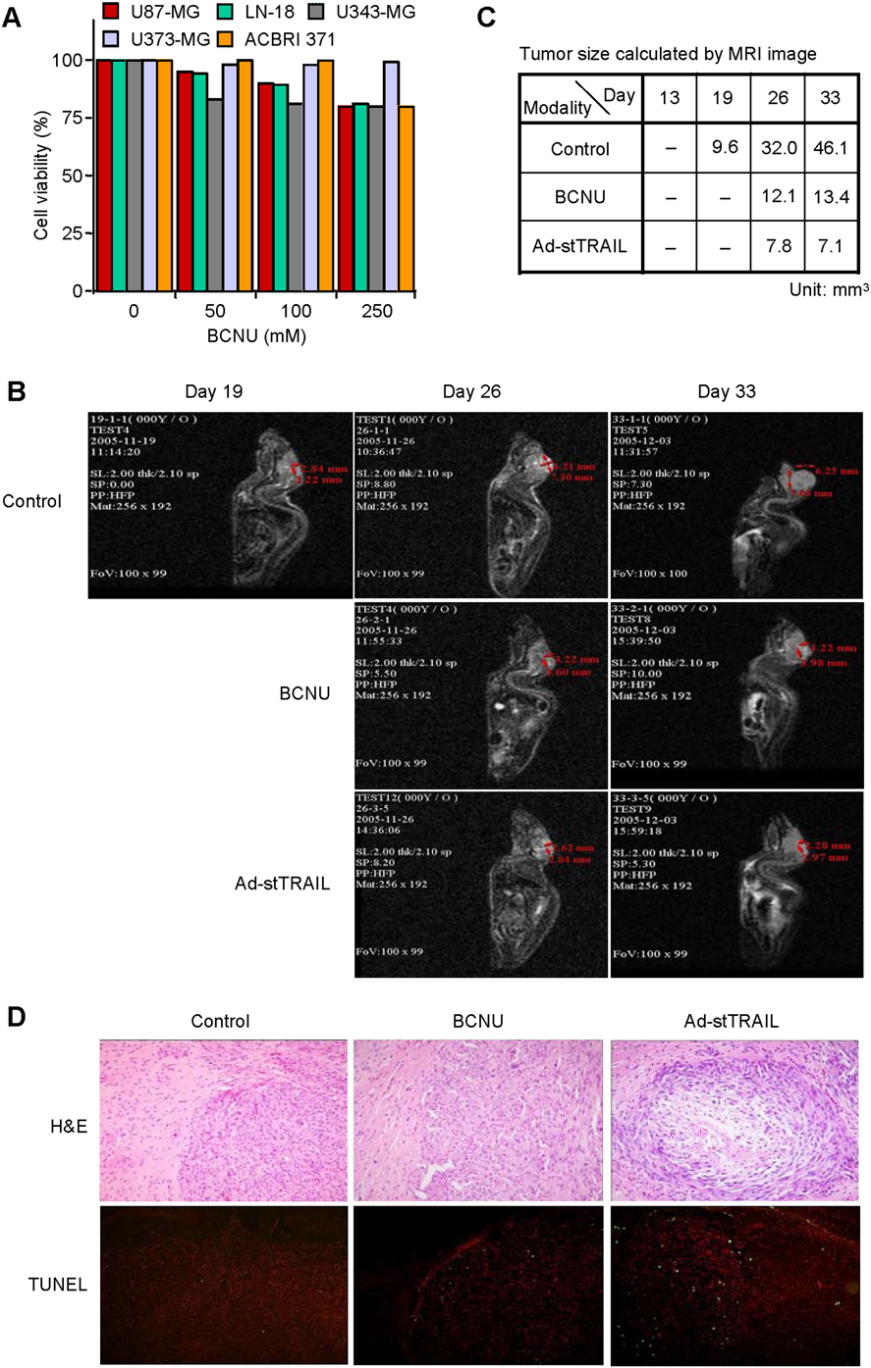
Comparison of Ad-stTRAIL with BCNU in Therapeutic Efficacy. (A) Susceptibility of glioma cells to BCNU *in vitro*. Cells were incubated with different concentration of BCNU. After three days, cells were processed for the analysis of cell viability. The results represent the mean for three separate experiments. The SE bars were omitted, but <5% at all the data points. (B) Representative MRI after treatment with saline control, BCNU or Ad-stTRAIL. U87-MG cells (1×10^5^ cells/10 µl per mouse brain) were stereotactically implanted into the right striatum of athymic mice (nu/nu). At day 14 and 18 after tumor implantation, saline or Ad-stTRAIL (1×10^10^ VP/head) was delivered intra-tumorally. BCNU (10 mg/kg) was intraperitoneally delivered at day 14, 16 and 18 after tumor implantation. Tumor growth was monitored by MRI at the indicated times (n = 14 for each experimental group; total n = 42). (C) Tumor size calculated by MRI shown in (B). (D) TUNEL staining and histology of tumor tissues after treatment with saline control, BCNU or Ad-stTRAIL. Twenty one days after intracranial implantation of tumors, mice were sacrificed and cryostat sections of brain were prepared. Cryostat sections were stained with H&E, or subjected to TUNEL staining.

We next compared the efficacy of Ad-stTRAIL with BCNU, a conventional therapy for malignant gliomas which crosses the blood-brain barrier. Although widely used for brain tumors, BCNU only moderately limited growth of glioma cells *in vitro* (Figure 7A). Compared to Ad-stTRAIL *in vivo*, BCNU was less effective in suppressing tumor growth (Figure 7B, C). Tumor tissue analysis after treating with BCNU or Ad-stTRAIL showed that Ad-stTRAIL was associated with more TUNEL positive cells than BCNU (Figure 7D).

**Figure 8:**
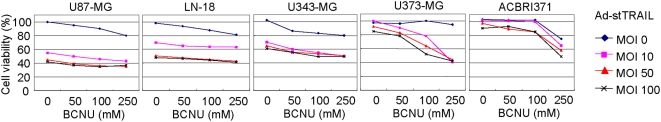
Susceptibility of glioma cells to Ad-stTRAIL, BCNU or Ad-stTRAIL+BCNU combination *in vitro*. Glioma cells, plated in 24-wells (1×10^5^ cells/well) in complete medium, were incubated with Ad-stTRAIL (0, 10, 50 or 100 MOI), BCNU (50, 100, 100 or 250 µM) or combination of Ad-stTRAIL and BCNU. Three days after incubation, cells were processed for the analysis of cell viability. The results represent the mean for three separate experiments. The SE bars were omitted, but <5% at all the data points.

**Figure 9:**
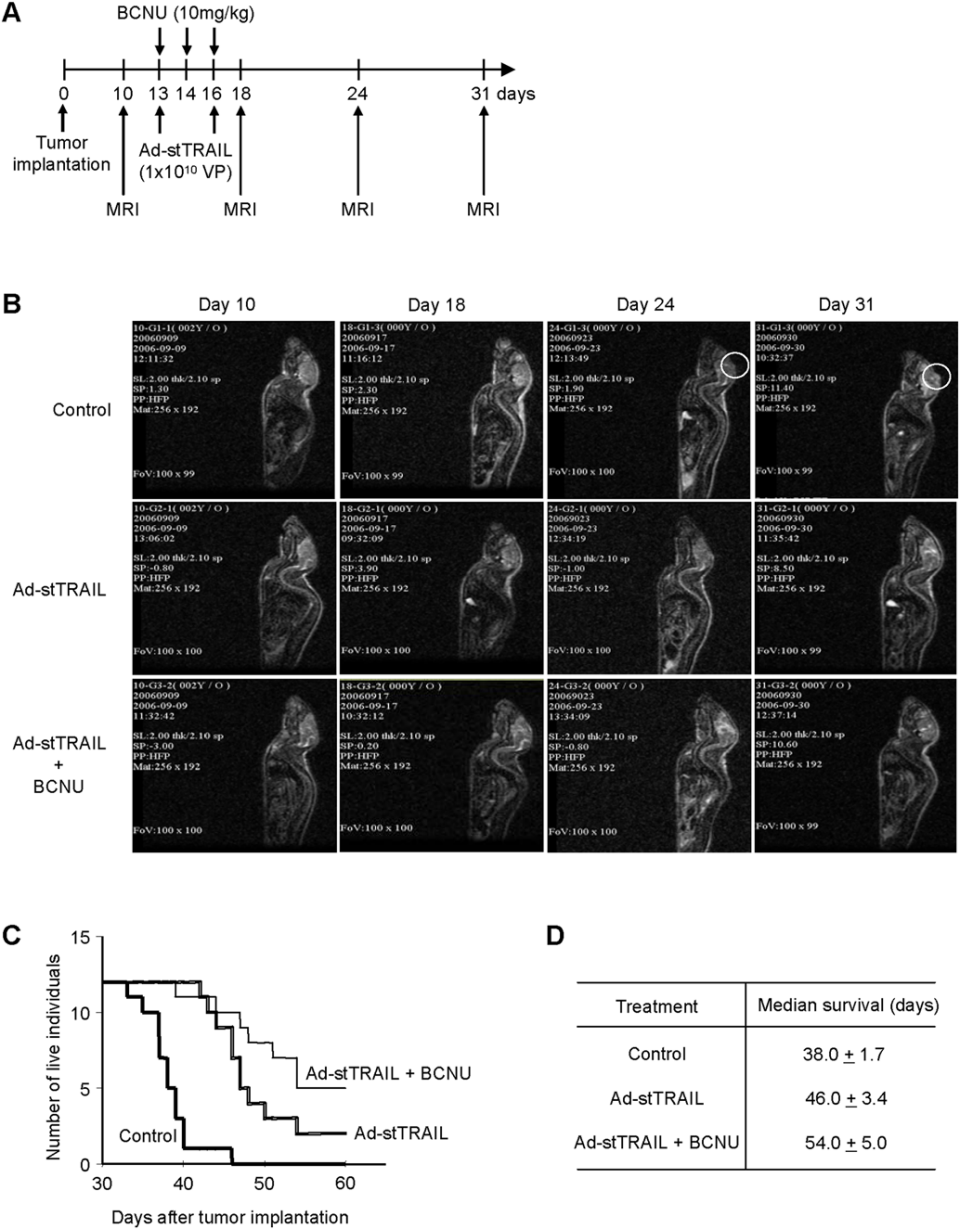
Comparison of Ad-stTRAIL+BCNU combination therapy with BCNU individual therapy in therapeutic efficacy. (A) The schematic drawing for experimental design and MRI analysis (see also Fig. 7B legend). (B) Representative MRI after treatment with saline control, Ad-stTRAIL individual therapy or Ad-stTRAIL+BCNU combination therapy (n = 15 for each experimental group; total n = 45; see also Fig. 3B legend). (C) Survival curve. Live animal were counted at the indicated times after treatment as described in (B) (n = 12 for each experimental group; total n = 36). (D) Median survival. The data described in (C) were analyzed. The range given for survival indicates ‘Anderson 95% CI’ in Kaplan-Meier survival estimates.

Generally, combination therapies yield better efficacy with fewer side effects, hence combination therapies are widely used in cancer treatment. We therefore evaluated the combination of Ad-stTRAIL and BCNU, *in vitro* and *in vivo*. BCNU modestly enhanced the apoptotic activity of Ad-stTRAIL in TRAIL-susceptible glioma cells including U87-MG, LN-18 and U343-MG (Figure 8). In contrast, BCNU significantly augmented cell death induced by Ad-stTRAIL in U373-MG and ACBRI371 (Figure 8), two tumor cell lines shown to be almost completely resistant to Ad-stTRAIL or TRAIL protein alone (Figure 2). Therefore, the combination of Ad-stTRAIL with BCNU broadens the range of tumor cells responsive to therapy. Finally, we evaluated therapeutic feasibility of Ad-stTRAIL/BCNU combination therapy *in vivo* (Figure 9). Ad-stTRAIL/BCNU combination therapy showed better tumor suppressor activity than Ad-stTRAIL individual therapy (Figure 9B). Ad-stTRAIL/BCNU combination therapy also improved the survival rate (Figure 9C). These results raise the possibility that combination therapy would be an effective strategy to treat even BCNU-resistant tumors.

Here, we demonstrate the therapeutic potential of a TRAIL-based gene therapy approach utilizing a construct designed to deliver a secretable form of trimerized TRAIL using a vector system for local delivery. Ad-stTRAIL vector showed better efficacy in an animal brain tumor model than BCNU. Combination therapy comprising Ad-stTRAIL and BCNU resulted in greater efficacy than Ad-stTRAIL or BCNU alone. Furthermore, Ad-stTRAIL was safe at the dose showing therapeutic efficacy. It is important to note that repeated intracranial injection of Ad-stTRAIL did not induce detectable anti-adenovirus immune responses. In fact, no neutralizing anti-adenovirus antibody was detectable in the plasma following repeated intracranial injection of Ad-stTRAIL (data not shown), suggesting that expected therapeutic efficacy can be obtained even after repeated delivery of Ad-stTRAIL. Although the mechanism for limited host immune responses to Ad-stTRAIL remains to be determined, the blood-brain barrier (BBB), separating the adenovirus-injection site from the blood circulation, seems likely to play a certain role. Taken together, our results raise the possibility that Ad-stTRAIL will be effective in other chemo-resistant tumors and serve as the basis for a clinical trail currently planned for malignant gliomas in Korea.

## Materials and Methods

### Reagents

Recombinant human TRAIL (rhTRAIL) was obtained from R&D systems (Minneapolis, MN). BCNU (Carmustin) was purchased from Sigma (St. Louis, MO). Antibodies against monoclonal human TRAIL was purchased from PeproTech (Rocky Hill, NJ).

### Cell culture

U87-MG, T98G, U373-MG, SG388, and LN-18 cells, originally obtained from American Type Culture Collection (ATCC) (Rockville, MD), were cultured in MEM, MEM, MEM, DMEM and DMEM, respectively. ACBRI371 cells, obtained from Cell Systems (Kirkland, WA), were maintained in DMEM. U343-MG cells, obtained from Dr. Chae-Ok Yun (Yonsei Cancer Center Hospital, Korea), were cultured in DMEM.

### Preparation of adenoviral vectors

An E1/E3-doubly deleted replication-incompetent adenoviral vectors Ad-stTRAIL or Ad-flTRAIL was constructed through Cre-lox recombination as described [20]. Ad-Ψ5 or Ad-flTRAIL propagated on 293 cells (ATCC) was purified by cesium chloride density gradient centrifugation and subsequent dialysis against Tris-saline buffer (0.14 M NaCl, 0.7 mM Na_2_HPO_4_, 0.025 M Tris, adjusted with HCl to pH 7.2). Ad-stTRAIL propagated on 293F cells (Invitrogen, USA) was purified using anion exchange chromatography and immobilized metal affinity chromatography. The purified virus was subsequently dialyzed against formulation buffer (10 mM Tris (pH 7.5), 75 mM NaCl, 5% (w/v) sucrose, 0.02% (v/v) Tween-80, 1 mM MgCl_2_, 100 µM EDTA, 0.5% (v/v) Ethanol and 10 mM L-Histidine). All viruses were kept at −80°C until use.

### Trans-well analysis

U373-MG cells were infected with adenovirus for 6 hours and transferred to the upper wells (Polycarbonate membrane 24 well, 0.4 µm pore, Corning) following two washes with HBS. U87-MG cells maintained at the bottom wells were co-cultured with the upper wells. Three days later, cell viability of U87-MG was analyzed by MTS assay using the MTS assay kit purchased from Promega (Madison, WI). rhTRAIL was used as a positive control.

### In vitro treatment with BCNU

ACBRI371, U87-MG, U373-MG, U343-MG or LN-18 cells, plated in 24-wells (1×10^5^ cells/well) in complete medium, were incubated with different concentration of BCNU (50, 100, 250 or 500 µM). Three days after incubation, cells were processed for the analysis of cell viability.

### Cell viability assays

Cells infected with adenoviral vectors or treated with rhTRAIL as a positive control were analyzed by MTS assay.

### Animal experiments for efficacy assessment

Athymic mice (nu/nu) were purchase from Charles River (Wilmington, MA) through a Korean distributor Orient Co., Ltd. To generate an orthotropic xenograft model for brain tumor, U87-MG human malignant glioma cells (1×10^5^ cells/10 µL per mouse brain) were stereotactically implanted into the right striatum of athymic mice using Just For Mice™ stereotaxic (Stoelting Co, IL). As an individual therapy, delivery of controls or Ad-stTRAIL to tumor sites was performed at the indicated times. BCNU (10 mg/kg) was injected intra-peritoneally followed by intra-tumoral injections of controls or Ad-stTRAIL. The changes of established tumor mass were detected by MRI. The survival profile is expressed as a Kaplan-Meier survival estimate. All the experiments done on animals were approved by an institutional review board.

### MR imaging

Mice were imaged following anesthesia with ketamin (57 mg/ml, 15 µl/10 g body weight, injected intra-peritoneally). MRI examinations were performed on a 1.5-T MRI system (GE Signa MR/i Hispeed Plus, GE Medical Systems, WI). The volume of tumor was calculated in T2-weighted axial, coronal and sagittal images.

### Safety evaluation of Ad-stTRAIL

5-week-old male and female rats purchased from Orient Co., Ltd. (Seoul, Korea) were given the control (the formulation buffer) or the modality (3×10^9^, 1×10^10^ or 3×10^10^ viral particles) at the putamen region of brain: exactly, 3.1 mm right, 0.4 mm front, 6.0 mm deep from the bregma. The same volume (30 µl/head) of Ad-stTRAIL formulation buffer was used as a vehicle control. Using 6 male and 6 female rats as an each experimental group, side effects, such as body weight loss, mortality and/or other general toxic symptom/appearance, were assessed.

### Detection of Ad-stTRAIL DNA in blood plasma, major organs or brain tissues

The tissues from organs or brain tissues from the virus injection site were weighed, homogenized and subjected to total DNA isolation using the QIAmp DNA mini kit (Qiagen). Blood, obtained from the tail vein or abdominal aorta, was mixed with heparin (50∶1) and centrifuged at 3,000 rpm for 10 minutes to separate plasma. The total DNA was isolated from the plasma using QIAmp DNA mini kit. The total tissue or blood DNAs were subjected to Taqman real time PCR (7900HT real time PCR, ABI) using the primer sets specifically amplifying the connecting junction region of between stTRAIL and the viral genome.

### Detection of stTRAIL protein

The tissues from the virus injection site were lysed in a buffer (25 mM Tris (pH 7.4), 50 mM NaCl, 0.5% Na-Deoxycholate, 2% NP-40, 0.2% SDS, 10% (v/v) protease inhibitor cocktail (Sigma)). After measuring protein concentration using the Bradford assay kit (Bio0Rad), lysates were subjected to ELISA. The plates (Immuno Maxisorp) purchased from Nalge Nunc (Rochester, NY) were prepared by coating with anti-human TRAIL antibody (1.5 µg/ml) in a binding solution (50 µl/well, 0.1M Na_2_HPO_4_ (pH 9.0)) and blocked with a blocking solution (200 µl/well, 2% BSA in PBS). The plates were then incubated with lysates or rhTRAIL as a standard. After 1 h incubation at 37°C, the plates were washed and treated with diluted biotinylated anti-human TRAIL antibody (100 µl/well, 0.2 µg/ml in incubation buffer) for 1 hr at room temperature. Following washing, diluted avidin-HRP (100 µl/well, 1/1000 dilution) was added and incubated for 30 min at room temperature. ABTS solution (Sigma) was added for color development and measured by reading absorbance at 405 nm.

### TUNEL staining and histology

Twenty one days after intracranial implantation of tumors, mice were sacrificed and cryostat sections of brain were prepared. Cryostat sections (6 µm) were air-dried and stained with H&E, or subjected to TUNEL staining. TUNEL staining was carried out using the kit DeadEnd Fluorometric TUNEL system from Promega (Madison, WI). Propidium iodide was used for counterstaining

### Statistical analyses

Analysis of differences was performed using the Wilcoxon rank sum test.
